# Knowledge, attitudes and intentions of the Syrian pregnant women toward labour analgesia, and its associated factors: a cross sectional study in Syria (2022)

**DOI:** 10.1186/s12913-023-10268-2

**Published:** 2023-11-09

**Authors:** Haidara Bohsas, Hidar Alibrahim, Sarya Swed, Yasmeen Abouainain, Mohamad Nour Nasif, Nagham Jawish, Mohammad Bani Almarja, Sara Aldarwish, Carol Ghareeb, Bisher Sawaf, Wael Hafez

**Affiliations:** 1 Faculty of medicine, Aleppo University, Aleppo, Syria; 2 Faculty of Medicine, University of Jordan, Amman, Jordan; 3 Damascus university Faculty of medicine, Damascus, Syria; 4 Department of Internal Medicine, Hamad Medical Corporation, Doha, Qatar; 5 NMC Royal Hospital, 16th Street, Khalifa City, Abu Dhabi, UAE; 6 Medical Research Division, Department of Internal Medicine, The National Research Centre, Cairo, Egypt

**Keywords:** Labour analgesia, Pregnant women, Labour pain, Syria

## Abstract

**Background:**

During the reproductive period among pregnant women, the worst pain a woman can experience is labour pain. Untreated labour pain has many detrimental effects on the mother and the fetus. Then, the inadequate levels of awareness and attitudes toward labour analgesia among pregnant women are considered a serious concern that influences no-healthy results for both the mother and the baby. Therefore, this research aimed to define the degree of Awareness, Attitude, and intent to use labour analgesia among pregnant women in Syria.

**Methods:**

We conducted a cross-sectional study from 7 September to 23 October 2022, in which we included Pregnant Syrian women aged 18 and above. The questionnaire was based on a prior study that included verified and validated scales, which consisted of 23 questions separated into four sections. The sample size was calculated using Fisher’s formula; however, our study included 638 participants. The data was analyzed using IBM SPSS Version 28.0, using descriptive and binary logistic regression methods.

**Results:**

Among those who had previous deliveries, 39.4% performed a caesarian delivery, and only 1.9% had a delivery at home. Nearly half of the study participants (50.4%) reported adequate knowledge about analgesia for obstetric pain. The inquired pregnant women who had children had more odds of knowledge than participants who had not. Respondents who were childbearing at the health center were more likely to have a good attitude (Adjusted Odds ratio = 4.728, P-value < 0.05, 95%CI: 1.035–21.589) than those who were childbearing at a national referral hospital. Also, the respondents above 31 years were less likely to desire labour analgesia than those aged 18–24.

**Conclusion:**

Our results revealed that Syrian pregnant women have a moderated awareness, attitudes, and desire regarding labour analgesia, indicating a serious health problem among this population group. It is recommended that local and global health organizations address the current condition relevant to this issue by implementing healthy educational programmes for Syrian women through coordination with obstetric and gynaecological professionals.

## Background

Labour is typically described as one of the most painful conditions in a woman’s reproductive life. Women’s descriptions of labour pain vary widely, from nonexistent to agonizing [1]. Labour pain consists of two components: visceral and somatic. The visceral type happens during the first stage of labour and is associated with the tension applied to the cervix. In contrast, the somatic type occurs at the end of the first stage and continues into the second stage and is caused by the pressure generated on the vaginal part of the cervix, the vagina, and the perineum [2]. Physical disturbances in women and infants, including hyperventilation, respiratory alkalosis, increased cardiovascular stress, fetal hypoxemia, and metabolic acidosis, have been linked to acute and severe labour pain episodes. Negative mental outcomes, including postpartum depression, PTSD, and difficulties interacting with the newborn, are potential negative health effects of labour pain [3, 4]. The World Health Organization (WHO) recommends various pain relief options for delivering women, including epidural analgesia, systemic opioids, relaxation techniques, and alternative treatments, such as massage, based on the pregnant women’s preferences [5]. Labour pain may be managed with various pharmacological and non-pharmacological approaches. In contrast, pharmacological therapies aim to eliminate the physical sense of pain using various drugs, and non-pharmacological measures enhance pain tolerance during delivery [6]. The American College of Obstetricians and Gynecologists (ACOG) considers pharmacological analgesia a safe intervention to relieve pain and physical stress that should be provided to every woman who requests it unless there is a medical contraindication to the intervention [7]. Compared to other pharmaceutical options for relieving labour pain, neuraxial labour analgesia (LNA) is superior in effectiveness and safety [8]. Minimal impacts on the maternal cardiovascular-pulmonary system and fetal physiology are expected from pain relief treatments. However, some research suggests that cesarean delivery rates rise dramatically when neuraxial analgesia is used, whereas other research suggests the opposite [9]. Health providers must inquire about every pregnant woman’s preference for pain relief during delivery as part of routine prenatal care. It has been shown that women who get continuous labour support, are sufficiently supplied with water, and are placed in an upright posture during the initial stage of labour need less pharmaceutical analgesia [10]. Women who have never given birth (nulliparous) could benefit greatly from more accurate prenatal education. This could help them have better birthing experiences and more realistic expectations since women who have never given birth before have reported more pain during birth. Researchers found that substantial clinical reductions in pain levels occurred after receiving childbirth education [11]. Women report feeling powerful and in control when allowed to make educated choices, particularly about managing and relieving pain during labour and after giving birth [12]. Many women in low- and middle-income countries do not have access to effective pain management because of a lack of knowledge and misunderstanding about the acceptability, safety, and availability of pain reduction choices [13]. Cultural and societal attitudes encouraging women to endure pain during labour and fearing that epidural medications would harm the unborn child were all considered barriers to analgesic use. As a result, all pregnant women should be required to learn about the benefits of labour pain relief in general and epidural analgesia in particular as part of their regular prenatal care [14, 15]. There is a lack of research on pregnant women’s knowledge and attitudes about labour analgesia in Syria. Therefore, this research aimed to define the degree of Awareness, preference, and intent to use labour analgesia among pregnant women in Syria.

## Methods

### Study design and date

We conducted an online cross-sectional study between 7 September and 23 October 2022.

### Study location

This study was conducted in all Syrian governorates, and all gynaecology and obstetrics hospitals and health community centres were available for data collection.

### Population

- Inclusion criteria:

The inclusion criteria were pregnant women of Syrian nationality aged 18 or older.

- Exclusion criteria:

Pregnant women under 18, non-Syrian nationality women, and participants who could not complete the survey due to a life-threatening obstetric condition were excluded from the study.

### Sample size calculation

The minimal sample size was detected by applying a single proportion of the population formula [n = [(Zα/2)2. P (1-P)]/d2]. With a 95% confidence level (Zα/2 = 1.96) and a 5% margin of error, P = the proportion of pregnant women who were aware of labour analgesia according to a previous study conducted in Ethiopia [16]. Moreover, after adding 5% to the non-response rate. The final requested sample size was 114.

### Sampling methods and techniques and data collection process

We inquired about the convenience and snowball methodologies to gather the sample study. Regarding the data collection process, fifteen collaborators were responsible for collecting the data. They distributed the printed questionnaires to the pregnant women in the obstetrics hospitals and clinics to fill out the survey; then, we saved the data on a safe Excel sheet file. Also, the questionnaire was uploaded to Google form and distributed via social media platforms, including Facebook, WhatsApp, and Telegram.

### Measures

The utilized scales were taken from a published cross-sectional study conducted in Ethiopia [16], in which a professional translator translated the questions from English to Arabic to confirm that the questions were readable for the participants. The questionnaire of this study included 23 questions separated into four sections. The socioeconomic characteristics and the obstetric status of the study population, while the second, third, and fourth parts measure the knowledge, Attitude, and Desire toward labour analgesia among Syrian pregnant women, respectively, as follows:

### Study tool

#### Socioeconomic characteristics variables

The sociodemographic information about participants was obtained in this section by asking them ten questions about their age, marital status, economic status, occupation, educational level, and residency place. In addition, this part investigated the obstetric status of women by asking them about the current gestational age, the mode and place of previous deliverers in case the women were multiparity, and the number of children they had. Nulliparous Women are those who have not delivered before, while parous women are those who have delivered at least for one time. Regarding the place of previous delivery, participants were asked if they gave delivery at a health center (a clinic that is part of a network of healthcare facilities staffed by general practitioners and nurses to offer medical services to the residents of a particular area), or national referral hospital (tertiary care centers, also known as referral hospitals, are specialized medical facilities that serve as a referral source for general hospitals in all major subspecialties. In certain instances, they may also provide secondary or primary care) [17].

#### Awareness of labour analgesia

Seven items were in this part to assess participants’ Awareness of labour analgesia. Participants were asked about their previous knowledge of labour analgesia, the sources of information about labour analgesia, the specific method for pain relief during labour they have heard about, their previous experiences with pain relief methods during delivery, and their satisfaction with prior experiences if it exists. Women who had an awareness of labour analgesia answered “yes” when asked about their previous information [18].

#### Attitude towards labour analgesia

Participants’ perceptions about labour analgesia were checked by asking them three questions in this part. Nulliparous women were asked about their expectations regarding labour pain, whereas multiparous women were asked to categorize it depending on its severity. A scale ranging from 0 to 10 was used to assess pain severity (0 = no pain, 5 = = moderate pain, 10 = = worst possible pain). In this domain, nulliparous and multiparous women were asked whether they believed labour pain should be relieved. When asked whether labour pain should be relieved, women who answered yes were considered to have a good attitude [18, 19].

#### Desire to have labour analgesia

To determine the participants’ Desire to have labour with no pain, they investigated three questions: their Desire for labour analgesia in their next delivery; if yes, “what is the method they prefer?“, and if they answered no, “what is the reason for pain relive refusing?“. Women who preferred to experience labour analgesia for their next delivery were considered to have a good desire [20].

### Pilot study

A survey was conducted online and distributed among 55 Syrian individuals to evaluate the questionnaire’s readability and clarity for potential respondents. The questionnaire was revised based on the feedback from participants, and Cronbach’s alpha was utilized to ensure the reliability and validity of the questionnaire, which was sourced from a previously published study conducted in Ethiopia.

### Ethical consideration

We obtained (IRB: SA**-**26,581) from the Syrian Ethical Society for Scientific Research after they gave their approval for this investigation. All procedures were conducted in accordance with the norms and standards in effect. Each participant gave their consent with knowledge. The survey needs approximately seven to ten minutes to be finished. All responses were stored securely in an online database. All participants were informed of the study’s purpose, the researchers’ identities, their ability to disengage from the study at any moment, the strict confidentiality of their data, and the fact that only completely reported data would be analyzed. Before beginning the online survey, participants were asked whether they were willing to complete the questionnaire after receiving a Google URL. During the distribution of the survey in hospitals, participants were asked if they were willing to participate. Before completing the questionnaire, participants are directed to a page comprising all relevant research materials.

### Statistical analysis

The data was examined using the IBM SPSS V. 28.0 package program (IBM Corporation, Armonk, NY, USA). *Statistical significance* was a P-value of less than or equal to 0.05. The quantitative data was given as a mean and standard deviation. In contrast, the categorical data was provided as frequency and percentage after validating that the data’s distribution was non-parametric after using the Shapiro-Wilk test. Finally, we used a binary logistic regression to estimate the anticipated values of “Odds ratios” to having an acceptable degree of awareness, attitude, and desire for labour analgesia between the dependent variable (awareness, and Attitude regarding labour analgesia) and independent factors (sociodemographic variables), which we based on the Ethiopian study’s cut off points.

Participants who answered ‘yes’ to the awareness question ' Do you have information about analgesics for obstetric pain?‘ were considered to have awareness. Participants who answered ‘yes’ to the attitudes question ‘Do you think the pain of childbirth should be relieved?‘ were considered to have good attitudes. Participants who answered ‘yes’ to the desire question ' Do you want to relieve labour pain in your next delivery?‘ were considered having a good desire.

## Results

### Socio-demographic information for the study participants

We invited about 719 pregnant women to participate in this study; however, just 638 were accepted, whereas 36 declined. Nearly half of the respondents (45.7%) had an age range of 25–31 years. Almost two-third of study participants (62.7%) belonged to secondary education. However, 32.9% of the participants were nulliparous. Regarding occupational status, about 60% of the participants were homemakers. Among those who had previous childbearing, 39.4% performed a caesarian section, and only 1.9% had childbearing at home. The majority of study participants, 96.2%, indicated the number of their family members between 0 and 4. 44.4% of pregnant in this study had gestational above 29 weeks. (Table 1)

**Table 1:** socio-demographic information for the study participants:

Variable	Categories	Frequency	Percentage
Age	18–24	222	32.5
	25–31	312	45.7
	> 31	149	21.8
Educational level	Primary	178	26.1
	Secondary	428	62.7
	University	1	1
	master	56	8.2
	No formal education	20	2.9
Partial status	nulliparous	225	32.9
	parous	458	67.1
Occupation	Housewife	409	59.9
	student	79	11.6
	Farmer	3	0.4
	Government employee	108	15.8
	other	67	9.8
	unemployed	17	2.5
Previous method of childbearing	Cesarean section	269	39.4
	normal	190	27.8
Place of birth	At national referral hospital	424	62.1
	At heath center	22	3.2
	At home	13	1.9
Place of residence	Countryside	358	52.4
	City	325	47.6
Number of family member	0–4	657	96.2
	5–9	26	3.8
Gestational age in weeks	1–12	149	21.8
	13–28	231	33.8
	> 29	303	44.4

### Pregnant females’ awareness of labour analgesia

Nearly half of the study participants (50.4%) reported they had information about analgesia for obstetric pain, whereas only 4.8% of them indicated that this information was from healthcare providers. 25.9% of respondents identified injection in the lower back (epidural, spinal) as one of the pain relief methods. However, more than half of the participants heard about childbirth pain relief during the current pregnancy. 33.1% of the study respondents showed experience with pain relief methods. (Table 2)

**Table 2:** Pregnant females’ awareness of labour analgesia:

Variable	Categories	Frequency	Percentage
Do you have information about analgesic for obstetric pain?	Yes	339	49.6
	No	344	50.4
information obtained from	experience in previous deliveries	86	12.6
	the media or reading	79	11.6
	antenatal talks in the hospital / maternal and child	71	10.4
	friends or relatives	70	10.2
	health care provider	33	4.8
methods of pain relief you heard about	Intramuscular injection in the thigh, shoulder and buttock	42	6.1
	Inhaled analgesia	17	2.5
	Injection in the lower back (epidural, spinal)	177	25.9
	Intravenous pethidine or morphine	59	8.6
	Massage, deep breathing and similar reassurance	44	6.4
When did you hear about childbirth pain relief?	During previous pregnancy	224	32.8
	During current pregnancy	354	51.8
	During previous child birth	105	15.4
Experience of pain relief methods?	No	457	66.9
	Yes	226	33.1
Which type of analgesia have you used before?	Lower back analgesia (subdural analgesia)	69	10.1
	Massage, deep breathing, reassurance	31	4.5
	Intramuscular injection (pethidine, diclofenac, or other)	63	9.2
	Intravenous (pethidine, tramadol, or other)	40	5.9
	Other	23	3.4

### Pregnant females believes and attitudes towards labour pain

Among participants with no previous birth experience, only 18.3% believed that labour pain is painful, while 67.2% accepted that labour pain should be relieved. However, only 66.6% agreed they would relieve labour pain in the next delivery (Table 3).

**Table 3:** pregnant women believes and attitudes towards labour pain:

Variable	Categories	Frequency	Percentage
Do you have previous birth experience?	No	232	34
	Yes	451	66
What are your expectations for labour pain (for women who have not yet given birth)	no idea	68	10
	pain free	7	1
	painful	125	18.3
Do you think the pain of childbirth should be relieved?	No	88	12.9
	Yes	459	67.2
	I don’t know	136	19.9
Do you want to relieve labour pain in your next delivery?	No	104	15.2
	Yes	455	66.6
	I don’t know	124	18.2

### Association between variables and awareness of labour analgesia

Table 4 shows the prediction of awareness toward labour analgesia among the study sample depending on the demographic variables. Parous participants had more odds of awareness than participants who were not (AOR = 1.739, p-value = 0.001, 95%CI: 1.258–2.403). (Table 4)

**Table 4:** association between variables and awareness of labour analgesia:

Variable	Awareness status		AOR(95%CI)	P - value
	Yes	No		
Occupation				
House wife	189	220	1	1
Student	41	38	2.794(0.967–8.073)	0.58
Farmer	2	1	2.224(0.717–6.905)	0.167
Government employee	59	49	1.200(0.088–16.439)	0.891
other	36	31	1.993(0.657–6.048)	0.223
unemployed	12	5	2.067(0.655–6.517)	0.215
Parity				
Nulliparous	91	134	1	1
Parous	248	210	1.739(1.258–2.403)	0.001
Age				
18–24	98	124	1	1
25–31	165	147	1.317(0.869–1.998)	0.195
> 31	76	73	0.928(0.628–1.371)	0.706
Previous method of childbearing				
Cesarean section	157	112	1	1
Normal	91	99	0.656(0.451–0.953)	0.562
Place of birth				
At national referral hospital	237	187	1	1
At heath center	7	15	0.420(0.124–1.419)	0.085
At home	4	9	0.979(0.222–4.306)	0.952

### Association between variables and attitude towards labour analgesia

Out of seven variables, only three predictors were statistically significant for predicting positive attitudes toward labour analgesia (p-value < 0.05). Childbearing participants had fewer odds of awareness than participants who were not (AOR = 0.127, 95%CI: 0.78 − 0.206). A high proportion of adequate attitude was noticed among participants, who indicated that the previous childbearing was normal compared to those with the last cesarean section (AOR = 2.009, 95%CI: 1.370–2.945). Regarding the place of birth, the respondents who were childbearing at the health centre were more likely to have a good attitude 4.728 times (95%CI: 1.035–21.589) more than those who were childbearing at a national referral hospital. (Table 5)

**Table 5:** association between variables and attitude towards labour analgesia:

Variable	Attitude status		AOR(95%CI)	P - value
	Yes	No		
Occupation				
House wife	135	274	1	1
Student	23	56	0.903(0.327-2.495)	0.844
Farmer	3	0	0.753(0.249–2.278)	0.615
Government employee	38	70	2,961,703,879(0)	0.999
other	21	46	0.995(0.341–2.902)	0.993
unemployed	6	11	0.837(0.273–2.567)	0.756
Parity				
Non- childbearing	21	204	1	1
Childbearing	205	253	0.127(0.78 − 0.206)	0.025
Age				
18–24	64	158	1	1
25–31	105	207	0.654(0.421–1.015)	0.0.58
> 31	57	92	0.819(0.546–1.228)	0.333
Previous method of childbearing				
Cesarean section	139	130	1	1
Normal	66	124	2.009(1.370–2.945)	0.000
Place of birth				
At national referral hospital	196	228	1	1
At heath center	7	15	4.728(1.035–21.589)	0.045
At home	2	11	2.567(0.444–14.822)	0.292

### Association between variables and desire for labour analgesia

The prediction of an adequate desire toward labour analgesia was statistically significant among two predictors (P –value < 0.05). The participants who were students had higher odds of desiring labour analgesia compared to housewives (AOR = 3.342, 95%CI: 1.257–8.889). The respondents above 31 years were less likely to desire labour analgesia than those aged 18–24 (AOR = 0.225, 95%CI: 0.121-0.418). (Table 6)

**Table 6:** association between variables and desire for labour analgesia:

Variable	Desire status		AOR(95%CI)	P -value
	Yes	No		
Occupation				
House wife	306	103	1	1
Student	29	50	3.342(1.257–8.889)	0.016
Farmer	3	0	0.652(0.227–1.877)	0.428
Government employee	71	37	1,817,409,198(0-)	0.999
other	34	33	2.159(0.769–6.059)	0.144
unemployed	8	9	1.159(0.399–3.366)	0.786
Parity				
Nulliparous	6	219	1	1
Parous	445	13	0.001(0-0.002)	
Age				
18–24	96	126	1	1
25–31	219	93	0.73(0.39 − 0.136)	0.000
> 31	136	13	0.225(0.121-0.418)	0.000
Previous method of childbearing				
Cesarean section	261	8	1	1
Normal	185	5	0.882(0.284–2.738)	0.828
Place of birth				
At national referral hospital	411	13	1	1
At heath center	22	0	0(0)	0.999
At home	13	0	1(0)	1

## Discussion

Pregnant women often experience moderate to severe pain during labour, a natural process. One of the important goals of Sustainable Development Goal 4 is to provide safe pain management during labour to improve delivery outcomes and maternal satisfaction. However, low awareness and positive attitudes towards labour analgesia among pregnant women can negatively impact the mother and fetus results. Therefore, this research aimed to assess the awareness, preference, and intention to use labour analgesia among pregnant women in Syria. This survey found that only a significant percentage of the interviewed Syrian pregnant women in the study were aware of labour analgesia (49.6%). Pain during labour and delivery is frequently accepted as a normal part of the birthing process in developing countries. It does not seem essential, and it certainly goes against the grain of traditional beliefs, to try to eliminate labour pain with medication. Studies conducted by Nabukenya (7%), Naithani et al. (9.5%), and Prakash et al. (7.14%) [18, 20, 21] produced results that cannot be compared to those of the current study. Although the results of our study are only relatively equivalent to those found in studies carried out by Mugambe et al. (56.3%), Minhas et al. (76%), and Karuga et al. (56%), it is possible that this may be due to the high level of education among the participants in the studies conducted by Karuga et al. and Minhas et al. Our findings differ from those obtained in research conducted in Uganda (87.8%), where a lower level of education was reported among the participants [18] and Ethiopia (74.1%) [16], but it was greater than those found in studies carried out in South Africa (48.3%) [22]. Most participants (67.2%) believed that labour should not include discomfort. In a survey of 225 women who had never given birth, 18.3% of respondents indicated that labour was unpleasant, while 10% of respondents claimed they had no idea what to anticipate from the pain of labour. Research conducted in India revealed 87% and 7.5%, respectively [19]. Most participants expressed a desire for labour analgesia in their subsequent birth despite having a relatively low level of awareness about painless labour. This aligns with the findings of a study conducted in Ethiopia, where 65.9% of participants expressed the same preference [16]. This was a lesser percentage than what was found in research conducted in Uganda (87.7%) and Nepal (72.2%) [18, 23], but it was a larger percentage than what was found in studies conducted by Prakash et al. (16.43%) [20] and Yadav et al. (13.5%) [24]. Regarding where people get their information regarding labour analgesia, the current study revealed that most participants obtained their knowledge about labour analgesia either from the experience of having previously delivered a child (12.6%) or through the media or books (11.6%). Although the research carried out in Ethiopia revealed that the majority of participants learned about labour analgesia through healthcare practitioners (27.3%), friends and family (24.2%), and other participants (24.2%) [16]. Other studies carried out in Uganda revealed that the majority of respondents obtained information from friends or family members (47%), while 26% received information from previous labour. Conversely, a study conducted in South Africa showed that the majority of respondents (56.5% or 55.3%) were women who had acquired information through previous experience or by consulting friends and relatives [25, 26]. Concerning the methods of pain relief, the majority of the participants reported that they had information about injections in the lower back (25.9%), either spinal or epidural. This was consistent with a study conducted in South Africa (32.9%). However, it was higher than the findings of a study conducted in Nigeria, which showed that only 10% of participants were aware of an injection in the back (epidural) [27]. Only a small percentage of participants (6.1%) were aware of intramuscular injections, compared to the much higher percentage of 65.9% in South Africa who were aware of the procedure [25]. The study revealed that individuals who had given birth previously (parous) were more likely to know the information than those who had not (AOR = 1.739, p-value = 0.001, 95%CI: 1.258–2.403). In Ethiopia, research showed a strong correlation between awareness of labour analgesia among parous women (adjusted odds ratio: 7.227, 95% confidence interval: 2.406–21.720) [16]; this was not the case for women who had not previously given birth. This could be because pregnant women have previous experience with the pain associated with labour, which prompted them to seek information regarding labour analgesics. Additionally, this study found that awareness regarding labour analgesia was not influenced by the educational status of the participants, which was consistent with the findings of a study by Deogaonkar et al [28]. This may be explained by the fact that most moms sought labour analgesia for their subsequent birth; if this were the case, then educational level and parity would not make a difference. This suggests that there was a low degree of awareness at all levels of the educational system. According to the study’s findings by Naithani and colleagues, there was no correlation between age and level of awareness [21]. This may be because women between the ages of 25 and 31 had a higher level of understanding than their counterparts.

### Limitations

This study excluded mothers in labour, which may have affected on demanding labour analgesia. An additional aspect that must be considered is the possibility that the survey results will be skewed because it was completed using an online questionnaire. We employed collaborators to collect responses using the non-probability snowball method. We also used the non-probability convenience method, as the collaborators collected data from the presence of Syrian women in hospitals or through the Internet, which is one of the most important limitations of online cross-sectional studies. This survey is administered to members of the community who are highly educated, have access to the internet via smartphones, and primarily reside in urban areas. In addition, the cross-sectional design does not provide evidence of a causal relationship.

## Conclusions

There was a moderate level of awareness, attitudes, and desire regarding labour analgesia among pregnant women. In Syria, there is a deficit of studies on pregnant women’s information, attitudes, and preferences toward labour analgesia. Barriers to analgesic usage include cultural and societal beliefs that encourage women to suffer through the discomfort of delivery and the idea that epidural medicines may harm the unborn child. Consequently, it is essential that all expectant mothers get education on the advantages of labour pain treatment in general and epidural analgesia in particular as part of the standard prenatal care they receive. When pregnant women get prenatal care, there is a need for a collaborative effort on the part of all parties involved in the healthcare system to change attitudes regarding labour analgesia, raise awareness of its availability, and satisfy women’s desire to have it.

## Data Availability

The authors have access to and have saved all of the data necessary to support this paper’s conclusion. All data are accessible upon reasonable request from the corresponding author.

## Declarations

### Data collection group

Haya Alkhlif Faculty of medicine .damascus university .syria (haya62694@gmail.com).

Lama Alazoak Faculty of medicine, Damascus university,Syria (Imyalazwq@gmail.com).

Mhd Almogtaba Alboga Factly of medicine, Damascus University, Damascus, Syria. (mogtabaalboga@gmail.com)

DANA MUNJJED Faculty of medicine,Damascus university,Damascus,Syria. (dmunjjed@gmail.com)

Youmna Hamoud Faculty of medicine,Damascus university,Damascus,Syria. (memosy1524@gmail.com)

RAMA BAYDA Faculty of medicine Damascus university, Damascus, Syria (wsambayda@gmail.com).

HUSSIN AL AHMAD Factly of medicine,Al furat university,Deir ezzor,Syria (alahmadhussain89@gmail.com).

Loujain kahoul Faculty of medicine, Damascus University, Assweidaa, Syria (Dr.loujainsy@gmail.com).
